# Ethanolic Fenugreek Extract: Its Molecular Mechanisms against Skin Aging and the Enhanced Functions by Nanoencapsulation

**DOI:** 10.3390/ph15020254

**Published:** 2022-02-20

**Authors:** Waleewan Eaknai, Phichaporn Bunwatcharaphansakun, Chutikorn Phungbun, Angkana Jantimaporn, Sasikan Chaisri, Suwimon Boonrungsiman, Ubonthip Nimmannit, Mattaka Khongkow

**Affiliations:** 1 National Nanotechnology Center (NANOTEC), National Science and Technology Development Agency, Khlong Luang 12120, Thailand; waleewan@nanotec.or.th (W.E.); phichaporn@nanotec.or.th (P.B.); chutikorn@nanotec.or.th (C.P.); angkana.jan@ncr.nstda.or.th (A.J.); sasikan.ch168@gmail.com (S.C.); suwimon@nanotec.or.th (S.B.); nubonthi@gmail.com (U.N.); 2 Faculty of Pharmaceutical Sciences, Chulalongkorn University, Bangkok 10330, Thailand

**Keywords:** fenugreek, rutin, anti-collagenase, anti-aging, collagen production, liponiosomes, nanoencapsulation, lipid particles, transdermal delivery, sustained release

## Abstract

Fenugreek, or *Trigonella foenum-graecum* L. (family Leguminosae) seeds, are typically used as food supplements to increase postnatal lactation. Fenugreek extract displays antioxidative and anti-inflammatory properties, but its mechanisms against skin aging have not been exploited. In this research, we are the first to define an in vitro collagenase inhibitory activity of fenugreek extract (IC_50_ = 0.57 ± 0.02 mg/mL), which is 2.6 times more potent than vitamin C (IC_50_ = 1.46 mg/mL). Nanoencapsulation has been applied to improve the extract stability, and subsequently enhanced its bioactivities. Liponiosome encapsulating fenugreek extract (LNF) was prepared using a high-speed homogenizer, resulting in homogeneous spherical nanoparticles with sizes in the range of 174.7 ± 49.2 nm, 0.26 ± 0.04 in PdI, and 46.6 ± 7.4% of entrapment efficiency. LNF formulation significantly facilitated a sustained release and significantly enhanced skin penetration over the extracts, suggesting a potential use of LNF for transdermal delivery. The formulated LNF was highly stable, not toxic to human fibroblast, and was able to enhance cell viability, collagen production, and inhibit MMP1, MMP9, IL-6, and IL-8 secretions compared to the extract in the co-cultured skin model. Therefore, ethanolic fenugreek extract and its developed LNF display molecular mechanisms against skin aging and could potentially be used as an innovative ingredient for the prevention of skin aging.

## 1. Introduction

Skin aging is a biological process induced by both intrinsic and extrinsic factors. Intrinsic aging is caused by changes in the elasticity of the skin over time, while extrinsic aging is attributed to UV radiation, pollution, and smoking. Exposure to UV radiation (photoaging) is a predominant factor [1,2]. The most important structures of the extracellular matrix (ECM) are collagen, elastin, and glycosaminoglycans (GAGs) [3]. Common features of skin aging include the generation of reactive oxygen species (ROS) and degradation of the ECM by overexpressed matrix metalloproteinases (MMPs). Cumulative oxidative stress and UV irradiation have been shown to increase the activity of degradative enzymes, especially collagenase and elastase [1,4,5]. This contributes to overall skin behaviors, including the loss of tensile strength and elasticity, and resultant wrinkling, and dryness. To cope with skin aging, research mainly focuses on the reduction of oxidative stress (antioxidant) and ECM degradation (anti-collagenase and anti-elastase) [6,7,8]. Natural products have attracted interest as a new generation of cosmeceuticals. There is numerous evidence that plants possess a variety of active compounds, for example, terpenoids, alkaloids, and phenolic compounds [9,10,11]. *Trigonella foenum-graecum* L. (fenugreek), belonging to the family Leguminosae, is a herbaceous plant that typically grows in western Asia, northern India, northern Africa, and the Mediterranean [12]. Fenugreek seeds are commonly used as a food, spice, galactagogue, and traditional medicine for diabetes [13,14,15]. Additionally, a toxicological study showed the safety of fenugreek seeds when used as a dietary supplement [16]. Fenugreek seeds contain numerous phytochemicals, including alkaloids, saponins, and flavonoids (rutin, quercetin, and vitexin) [17]. Many studies reported fenugreek’s antidiabetic, anticancer, antimicrobial, antioxidative, and anti-inflammatory activities [18,19,20,21,22,23]. Rutin is a compound of fenugreek that has been investigated for its anti-aging and antioxidant activities, as well as inhibition of collagen degradation [24,25,26]. Although there is no report implicating fenugreek extract in cosmeceuticals, it is expected to display biological properties that show these potential functions. Nevertheless, the general issues of using natural extract in cosmetic products are poor penetration into the skin, solubility, and stability. Nanoencapsulation is a technique that has been used to (i) improve stability, (ii) increase water solubility, (iii) enhance skin permeation and absorption, and iv) slowly release active ingredients [27,28]. There are many different types of nanoparticles, such as liposomes, niosomes, and liponiosomes (a combination of liposomes and niosomes). Liponiosomes are composed of phospholipid and non-ionic surfactants that enhance the capability and control the release of nano-carriers [29]. The morphology of liponiosomes resembles that of liposomes. However, liponiosomes are more flexible and adaptable and, importantly, function differently. The advantages of liponiosome carriers are that they are deformable to penetrate pores that are much smaller than the carrier’s size and have the capability to transport both water and fat-soluble agents. Hence, liponiosomes are promising carriers for delivering an active ingredient into the skin [30]. In this study, a fenugreek extract using rutin as an analytical marker was validated and identified. The biological activities of fenugreek extract were elucidated. Fenugreek has shown anti-collagenase activity and increased collagen production, which can refer to the anti-aging property. Liponiosome encapsulating fenugreek extract (LNF) was developed and characterized. Cytotoxicity of LNF was observed in human dermal fibroblasts using cell-based assays. These also included an investigation of molecular mechanisms on LNF anti-aging properties. The results suggested that LNF is non-toxic to dermal fibroblast cells and can inhibit the secretion of MMP1, MMP9, IL-6 and IL-8 upon UV-induced skin aging. Thus, our findings support the implication of fenugreek extract and LNF as innovative anti-aging agents, highlighting their potential as active ingredients in cosmeceutical products.

## 2. Results

### 2.1. UHPLC Validation and Identification of Rutin in Fenugreek Extract

**Figure 1:**
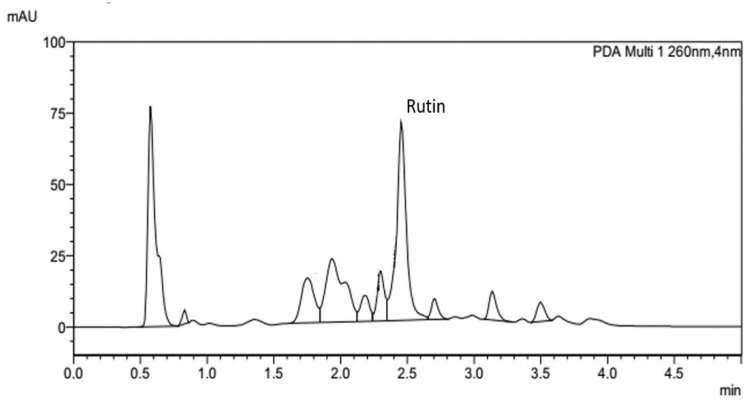
Chromatogram of ethanolic fenugreek extract using rutin as a chemical marker.

After ethanol maceration, the oily yellowish–brown paste of fenugreek extract was obtained. The identification of rutin content as an analytical marker was investigated using UHPLC. Optimum separation of rutin from fenugreek extract was achieved with a retention time of approximately 2.458 min (Figure 1), and was confirmed by using LC-MS with the detected product ion at 301 (Supplementary Figure S1). The validation of rutin was performed using AOAC guidelines [31,32] and is shown in Table 1. The equation in the table represents good linearity with a coefficient of determination (r^2^) of 0.9998, with accuracy measured by the percentage of the recovery. Sensitivity was indicated using Limit of Detection (LOD) and Limit of Quantitation (LOQ) at 5.17 and 15.67 µg/mL, respectively. The fenugreek extracts were prepared at various concentrations and the amount of rutin in the extract was interpreted using the area under the curve, relative to the standard curve of the commercial rutin reference. Results showed that the prepared extract contains 7.73 ± 0.40 mg/g of rutin.

### 2.2. In Vitro Collagenase Inhibition and Collagen Production of Fenugreek Extract

**Figure 2:**
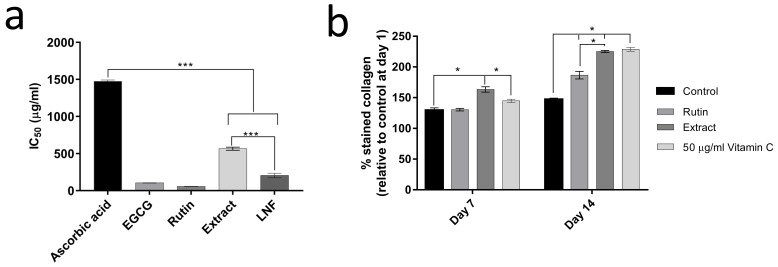
Anti-aging activities’ evaluation of fenugreek extract: (**a**) In vitro collagenase inhibition of fenugreek extract (denoted as Extract) and liponiosome encapsulating fenugreek extract (denoted as LNF). (**b**) Effect of fenugreek extract on collagen production. Human dermal fibroblast cells were treated with 125 µg/mL of fenugreek extract and rutin, and 50 µg/mL of vitamin C as a positive control and 0.005% DMSO as a control (vehicle), for 7 and 14 days. Data are reported as means ± SD (*n* = 3). *, *p* < 0.05; ***, *p* < 0.001.

An anti-aging activity of fenugreek extract was firstly identified by in vitro collagenase inhibitory assay. We observed that the IC_50_ of rutin trihydrate standard, fenugreek extract, and epigallocatechin gallate (EGCG) as a positive control was 0.06 ± 0.01, 0.56 ± 0.02, and 0.11 ± 0.01 mg/mL, respectively (Figure 2a,b). Notably, the inhibitory concentration of fenugreek extract was significantly lower than ascorbic acid (IC_50_ = 1.46 ± 0.02 mg/mL), indicating that fenugreek extract and rutin trihydrate standard exhibit anti-collagenase activity. The ability of fenugreek extract on collagen production was next investigated using human dermal fibroblast. Cells were treated with 125 µg/mL of extract and vitamin C as a positive control for 7 and 14 days. Cells were stained with Sirius Red and stained collagens were dissolved with NaOH. As shown in Figure 2b, the Sirius Red represents the collagen content. The amount of stained collagen was significantly enhanced in extract-treated cells (163% and 131%) compared to a vehicle control (225% and 187%) at day 7 and day 14, respectively. This induction was similar to the effect of vitamin C-induced collagen production and was significantly higher than rutin-induced collagen production in human fibroblast cells. This result demonstrated that fenugreek extract induces collagen production in human dermal fibroblast cells.

### 2.3. Physicochemical Characterizations of Formulated LNF

**Figure 3:**
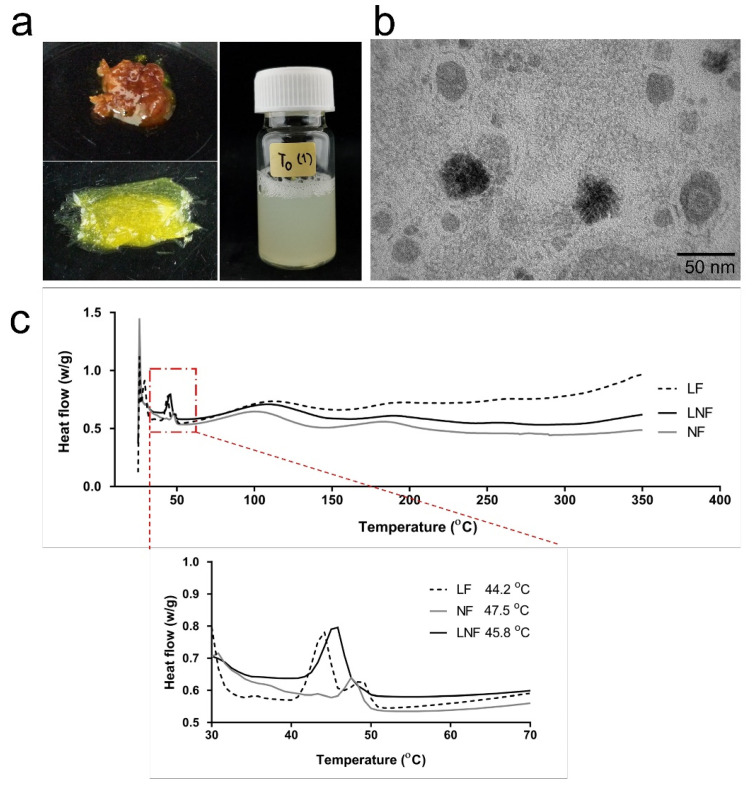
Physical appearances of fenugreek extract and liponiosome encapsulating fenugreek extract (LNF): (**a**) (Upper left) fenugreek extract form, (lower left) LNF paste form, and (right) 1% in water solution. (**b**) Physicochemical characterization of formulated nanoparticles showing a transmission electron microscope (TEM) image of LNF. (**c**) Physicochemical characterization of formulated nanoparticles showing a differential scanning calorimeter (DSC) graph of LNF compared to LF and NF.

To improve fenugreek extract stability and improve its appearance, formulation of LNF was performed using all ingredients demonstrated in Table 2, resulting in a yellow–gold paste substance (Figure 3a: lower left) which is easily dispersed in water to obtain a yellowish solution (Figure 3a: right).

The LNF displayed a miscellaneous and soft herbal scent. The physicochemical characterization of formulated LNF was investigated using dynamic light scattering (DLS) and is shown in Table 3. The particle size had a range of 174.7 ± 49.2 nm, with a low polydispersity index (PdI = 0.26 ± 0.04) and negatively charged surface due to phospholipids [33]. The encapsulation and loading efficiencies were approximately 46.6 ± 7.4% and 33.5 ± 4.0%, respectively.

A micrograph shows the morphology of formulated particles as a spherical particle encapsulating fenugreek extract inside Figure 3a, with diameters in the range of 50–150 nm (Figure 3b). Endothermic peaks in the DSC thermogram (Figure 3c) located at 45.8 °C represent the melting temperature of LNF, which is between the melting temperatures of liposome at 44.2 °C and niosome at 47.5 °C, respectively. The morphology and thermal behavior could confirm that LNF displays the physical characteristics of both liposomes and niosomes.

**Figure 4:**
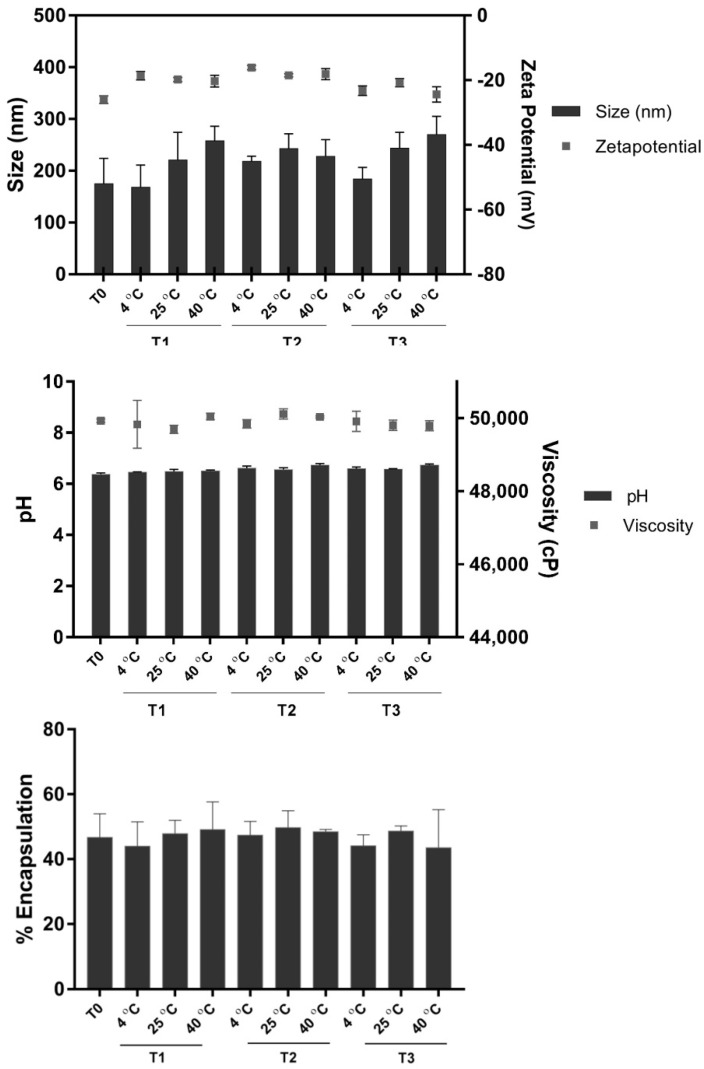
Stability data of LNF over 3 months (mean ± SD), in term of size, zeta potential, pH, viscosity, and % encapsulation of fenugreek extract.

LNF was kept at 4 °C, 25 °C, and 40 °C for 3 months to study its stability in terms of particle size, PdI, and ζ potential using the DLS technique. The pH and viscosity of the obtained particles were also investigated. We observed a slight increase in the particle sizes stored at 40 °C compared to 4 °C, but these changes were not statistically different. Additionally, there was no significant change in the other characteristics of formulated LNF, including pH, viscosity, and % encapsulation. These results all suggested that the obtained particles were physiochemically stable (Figure 4).

### 2.4. Releasing Profile and Skin Penetration of Formulated LNF

**Figure 5:**
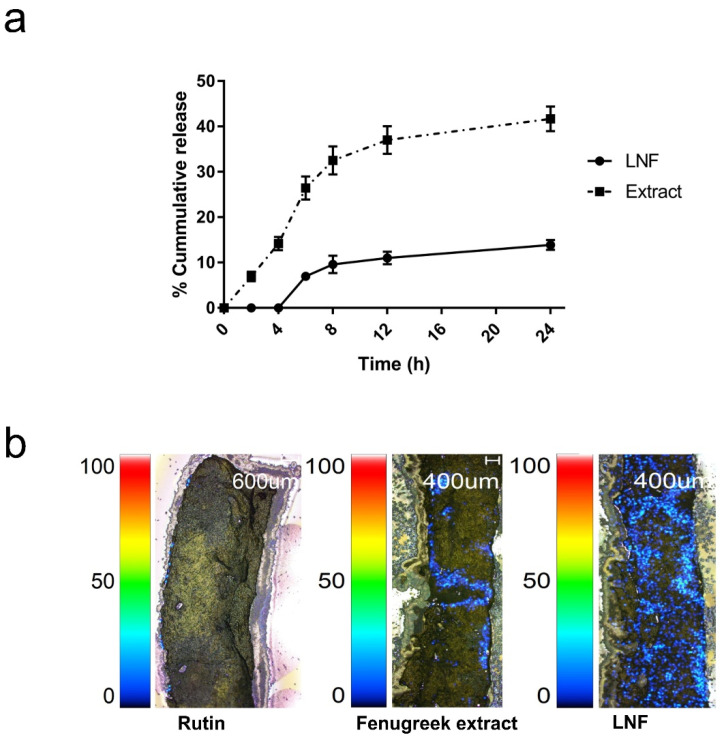
The releasing profile and skin penetration of formulated LNF: (**a**) In vitro releasing profiles of LNF within 24 h using Franz diffusion cells. (**b**) Porcine skin permeabilization. The IMS image showed the cumulative amount of rutin (blue) permeated through porcine skin, which was treated with rutin standard, fenugreek extract, and LNF for 24 h. Rutin was detected at 609 m/z.

The releasing profile (Figure 5a) demonstrated that fenugreek extract was released simultaneously, whereas its liponiosomes were detected 2 h later as 6.98 ± 0.37% and increased to 23.92 ± 1.41% at 24 h, suggesting the sustained release behavior of LNF. In order to investigate the potency of LNF on transdermal delivery, skin penetration was performed using ex vivo porcine skin. Imaging mass microscope (IMS) analysis was employed to observe a cumulative amount of rutin that transported across porcine skin of rutin standard, the extract, and LNF. In Figure 5b, we observed that fenugreek extract and LNF showed a higher amount of accumulated rutin on the skin compared to rutin standard. This result indicated that the permeability of fenugreek extract and LNF was greater and deeper than rutin at 24 h. Additionally, skin penetration of formulated LNF was noticeably enhanced compared to those of extract. According to the releasing profile and ex vivo skin permeation study, LNF possibly enhances the efficiency of the extract by increased permeation and controlled release.

### 2.5. Effect of LNF and Fenugreek Extract on Cell Viability and Collagen Production in Human Dermal Fibroblast Cells

**Figure 6:**
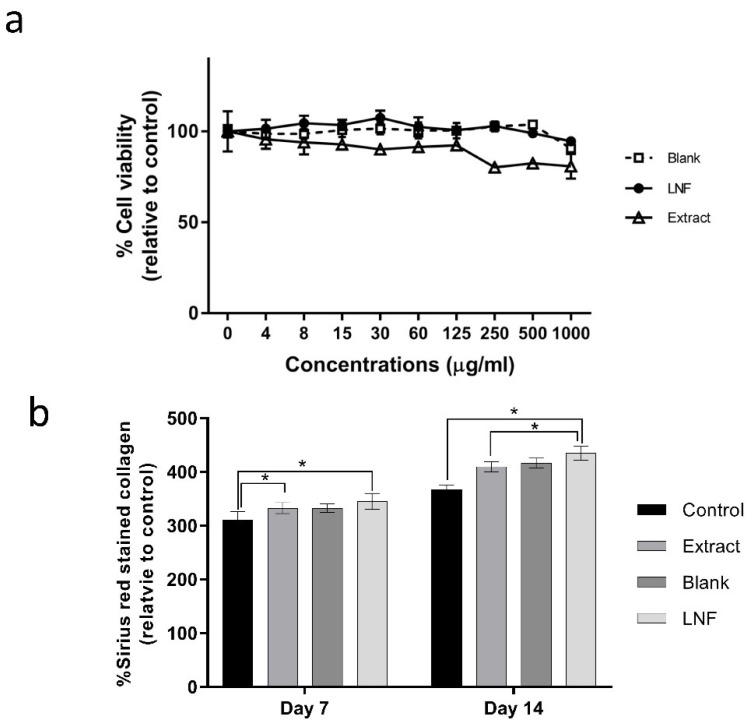
Evaluation of LNF activities: (**a**) Cytotoxicity induced by LNF: The effect of blank, LNF, and fenugreek extract on cell viability. Human dermal fibroblast cells were treated with different concentrations of blank (liponiosome without fenugreek extract denoted as Blank), LNF, and fenugreek extract for 24 h. Data are means ± SD (*n* = 3). (**b**) Collagen production induced by LNF: The effect of LNF on collagen production. Human dermal fibroblast cells were treated with 0.005% DMSO as a control (vehicle), 7 µg/mL of extract, and 100 µg/mL of LNF (7 µg/mL of extract equivalence), together with blank particles, for 7 and 14 days. Data are represented as means ± SD (*n* = 3). *, *p* < 0.05.

To investigate the cytotoxicity of formulated LNF, cells were treated with blank, LNF, and fenugreek extracts at 0–1000 µg/mL (twofold dilution) for 24 h. Cell viabilities were measured using an MTT assay. As shown in Figure 6a, the cell viability decreased in extract-treated cells, whereas no effect was observed in blank and LNF-treated cells. The data indicated that LNF showed a lower toxicity than extract. We further investigated the ability of LNF on the induction of collagen production in human dermal fibroblast cells. Cells were treated with 7 µg/mL of extract and 100 µg/mL of LNF (7 µg/mL of extract equivalence), together with blank particles as a control, for 7 and 14 days. As shown in Figure 6b, for treatment with LNF, the amount of stained collagen was increased by 30% and 50% at 7 and 14 days, respectively, compared to extract treatment. Additionally, in Figure 2a, the formulated LNF enhanced anti-collagenase activity, as IC_50_ was significantly reduced to 0.21 ± 0.03 mg/mL compared to fenugreek extract (0.57 ± 0.02 mg/mL). These results demonstrated that the formulation of LNF could potentiate an induction of collagen production in human dermal fibroblast cells and enhance the anti-collagenase activity of fenugreek extract. It is possible that nano-formulation of fenugreek extract significantly improves its potency as an active ingredient in anti-aging products.

### 2.6. Role of Formulated LNF on MMP1, MMP9, IL-6, and IL-8 Inhibition after UV Exposure

**Figure 7:**
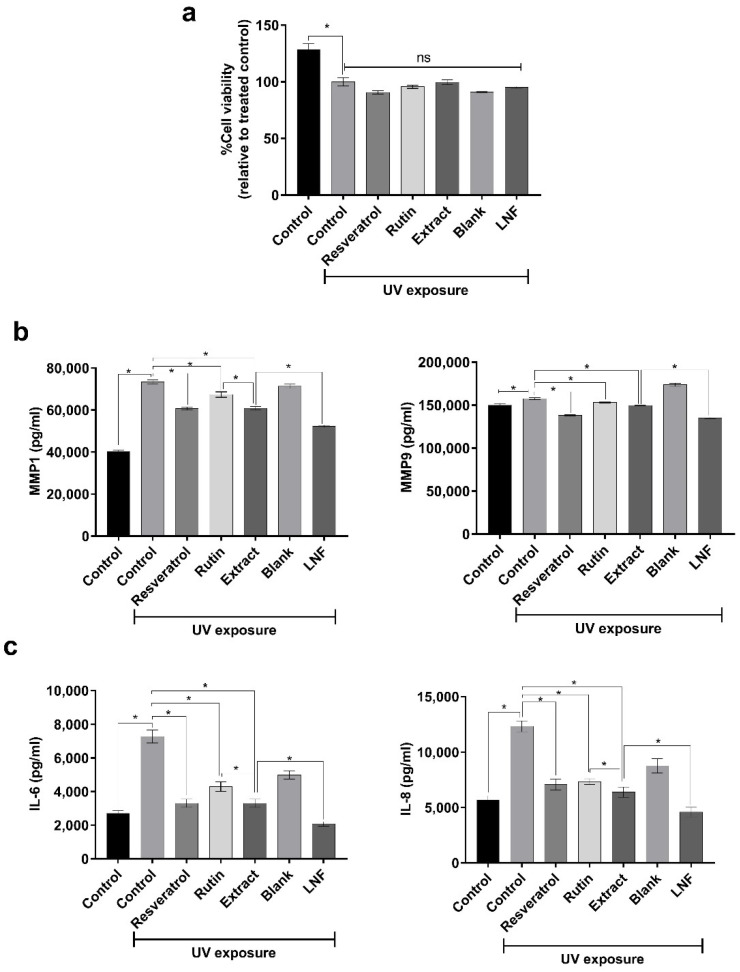
Inhibition of UV-induced MMPs and interleukin secretion on co-cultured skin cells by fenugreek extract and LNF: (**a**) Effect of UV-induced cytotoxicity after the pretreatments of resveratrol, rutin, fenugreek extract, blank nanoparticles (liponiosome without fenugreek extract), LNF nanoparticles, and 0.005% DMSO as a control (vehicle). HaCAT and human dermal fibroblast cells were co-cultured and pretreated with 7 µg/mL of extract and 100 µg/mL of LNF (7 µg/mL of extract equivalence), together with 100 µg/mL of blank, 10 µg/mL of resveratrol, 7 µg/mL of rutin as a positive, and 0.005% of DMSO as a control (vehicle), for 24 h before UV exposure. Data are means ± SD (*n* = 3). *, *p* < 0.05. (**b**) The levels of UV-induced MMP1 and MMP9 secretions after fenugreek extract and LNF treatments. Data are reported as means ± SD (*n* = 3). *, *p* < 0.05. (**c**) The levels of UV-induced IL-6 and IL-8 secretions after fenugreek extract and LNF treatments. Data are reported as means ± SD (*n* = 3). *, *p* < 0.05.

We next investigated some possible mechanisms of LNF on anti-aging activity. UV exposure is one of the main regulators of skin aging, as it has been reported to induce the expression of certain members of the matrix metalloproteinase (MMP) family, which causes collagen collapse, including MMP1, MMP3, and MMP9 [34,35]. We observed that UV exposure was highly toxic to co-cultured skin cells (Figure 7a) and enhanced the production of MMP1 and MMP9 cytokines (Figure 7b). In addition, the treatments of fenugreek extract and formulated LNF were able to significantly reduce the secretions of MMP1 and MMP9 compared to control cells after UV irradiation. These results were consistent with the treatment of rutin and resveratrol as an anti-aging agent control. Notably, the reduction of MMP1 and MMP9 expressions in LNF treated cells was significantly lower than in the extract treatment. Hence, LNF could reduce the secretions of MMP1 and MMP9 upon UV exposure, and subsequently prevent photo-induced skin aging.

Additionally, the exposure of UVR on human skin can also mediate an induction of the inflammation and pro-inflammatory cytokine activation, such as TNF-α and interleukins, including IL-1β, IL-2, IL-6, and IL-8 [36]. We then examined the function of LNF on the inhibition of UV-stimulated inflammatory cytokine production, including IL-6 and IL-8. In Figure 7c, we observed that UV irradiation significantly enhanced the production of IL-6 and IL-8 cytokines in co-cultured skin cells. A significant decrease in IL-6 and IL-8 expressions were observed with fenugreek extract treatment compared to control. Interestingly, the treatment of fenugreek extract exhibited considerably higher IL-6 and IL-8 inhibitions in comparison with rutin, whereas these inhibitory activities were similar to resveratrol-treated groups. Notably, the expressions of IL-6 and IL-8 in LNF-treated cells were significantly lower compared to fenugreek extract group after UV exposure. Together, these results indicated that LNF could potentially inhibit UV-mediated IL-6 and IL-8 production, and subsequently prevent photo-stimulated skin inflammation and skin aging.

As a consequence, these results suggest that ethanolic extract of fenugreek and its nano-formulation could potentially be used as active ingredients for the prevention of aging.

## 3. Discussion

Fenugreek extract has been demonstrated to possess antioxidant, antiradical, and anti-inflammatory activities in several studies [29,37], though its anti-wrinkle property has still not been exploited. This research was the first to identify an anti-aging activity of fenugreek extract using in vitro collagenase inhibitory assay. Prior studies have suggested the use of plant extracts with anti-collagenase activity as an active ingredient in cosmeceutical products such as grape pomace extract [38], green tea extract [39], and mushroom extract [40]. Moreover, the effect of fenugreek extract on collagen production in human dermal fibroblast cells has never been reported. Tamara et al. (2006) has demonstrated the effect of flavonoids on collagen synthesis in human fibroblasts using rutin, as an active compound of fenugreek extract, that helps to increase collagen [41]. This study of fenugreek showed its promising anti-aging capacity and represented rutin as a biological marker.

Rutin (3′,4′,5,7-tetrahydroxy-flavone-3-rutinoside) was used in this study as it is a flavonol glycoside that has been reported in several plants, including *Fagopyrum esculentum Moench*, *Ruta graveolens* L., *Sophora japonica* L., *Eucalyptus* spp. [37], and fenugreek [17]. Rutin possesses antioxidant, cytoprotective, anticarcinogenic, neuroprotective, and cardioprotective activities, proposing its application in the prevention of aging and aging-related diseases. Our results showed that the prepared extract displayed a high amount of rutin content, which was hardly found or detected as a component of fenugreek extract in other studies. Most other studies have reported trigonelline as a major chemical constituent of fenugreek extract [18,37,38,42,43,44,45,46,47].

Even fenugreek extract possesses a potential activity as an anti-aging agent. Its color and stability are still a challenge for this application. Nanoencapsulation has been reported as a specific technology that is able to stabilize, mask the odor, enhance water solubility, control the release of biological compounds [48,49], and improve the physical appearance of natural products. Liponiosome has been applied in this study due to its unique properties, which are composed of hydrophilic and hydrophobic moieties, that can encapsulate a wide range of substances with different solubilities [50]. We formulated fenugreek extract encapsulated liponiosomes (LNF), which is a hybrid carrier comprising liposomes and niosomes characteristics. The hybrid components of the developed LNF were confirmed by the melting temperature using DSC, reflecting both the thermal characteristics of liposomes and niosomes.

The stratum corneum is an outer layer of skin that works as an effective barrier against harmful substances by restricting their transportation across the skin. However, the use of nanocarriers has been proved to improve transdermal delivery by enhancing the penetration of drugs or substances through this barrier [51]. To investigate the potency of LNF on transdermal delivery, ex vivo procine skin penetration has been employed as its anatomical characteristics are mostly similar to those of human skin, in terms of skin thickness, follicular structure, and hair density [52]. Our results show that the permeability of LNF was greater and deeper than fenugreek extract and rutin, and the releasing profile of LNF had a slower release than fenugreek extract, suggesting the formulated LNF was noticeably superior to those of extract on skin penetration. Additionally, the formulated LNF enhanced anti-collagenase activity, as IC_50_ was significantly reduced to 0.205 ± 0.03 mg/mL compared to fenugreek extract (0.567 ± 0.02 mg/mL). These results demonstrated that the formulation of LNF could potentiate an induction of collagen production in human dermal fibroblast cells and enhance the anti-collagenase activity of fenugreek extract. Nano-formulation of fenugreek extract potentially helps improve its potency using it as an active ingredient for the prevention of aging.

Exposure to UV radiation is the main factor of extrinsic skin aging, known as pre-mature aging or photoaging [53,54]. The radiation causes the activation of cell surface receptors of skin keratinocytes and fibroblasts, leading to an induction of dermal extracellular matrix expressions, especially the matrix metalloproteinases family (MMPs). It has been demonstrated that chronic exposure to low doses of UVA causes an upregulation of mRNA levels of collagenase-1 (MMP1), stromelysin-1 (MMP3), and gelatinase A (MMP2) [55]. As a consequence, the degradation of skin collagen elastic fibers, as well as a shutdown of new collagen synthesis, occurs. This phenomenon affects the integrity, elasticity, and structures of the skin, after the deregulation of skin protective functions, accounting for wrinkle formation and signs of skin aging [54,56]. In addition, the exposure of UVR on human skin mediates an expression of TNF-alpha, which is an important regulator of inflammatory cascade in skin. UV irradiation also initiates the activation of both inflammation and pro-inflammatory cytokine secretions, such as interleukin-2 (IL-2) and interleukin-6 (IL-6) [36]. As a result, this UVR-induced inflammatory response has an important role on the induction of burned skin with signs of redness, irritation, and erythema [35,57,58,59].

Hence, to prevent the cause of premature aging, the finding of some novel active agents with these preventive functions would be ideal. Our results demonstrated that the formulation of LNF could potentiate an induction of collagen production in human dermal fibroblast cells, could reduce the secretions of MMP1, MMP9, IL-6, and IL-8 upon UV exposure, and could enhance the anti-collagenase activity of fenugreek extract. Therefore, an ethanolic fenugreek extract and its nano-formulation could be of potential use as a novel active ingredient in cosmeceutical products and that nanoencapsulation is able to enhance the function and activity of fenugreek extract as an anti-aging agent.

## 4. Materials and Methods

### 4.1. Plant Materials and Chemical Reagents

Fenugreek seed powder was received from Herbal Acharn’s Home Co., Ltd. (Bangkok, Thailand). Rutin trihydrate (>98% purity), tricine buffer, collagenase from clostridium histolyticum and FALGPA (N-[3(2-furyl) acryloyl]-Leu-Gly-Pro-Ala), direct red 80, picric acid, and dimethyl sulfoxide were purchased from Sigma-Aldrich (St. Louis, MO, USA). Acetonitrile, ethyl acetate, absolute ethanol, formic acid, sodium dehydrate phosphate, disodium hydrogen phosphate, hydrochloric acid, and sodium hydroxide were purchased from Carlo Erba (Emmendingen, Germany). Commercial grade ethanol was purchased from Italmar Co., Ltd. (Bangkok, Thailand). Cholesterol was purchased from Cosmeplus Co., Ltd. (Bangkok, Thailand). Propylene glycol and solubilizer mixture were purchased from S. Tong Chemicals Co., Ltd. (Nonthaburi, Thailand). Sorbitan oleate was purchased from Croda Co., Ltd. (Bangkok, Thailand). Phospholipid (Phospholipon 90G) was purchased from Cargill Siam Ltd. (Bangkok, Thailand). Tocopherol acetate was purchased from Namsiang Co., Ltd. (Bangkok, Thailand). The preservative was purchased from Forecus Co., Ltd. (Bangkok, Thailand). Paraformaldehyde was purchased from Himadia Laboratories (Mumbai, India) and 3-(4,5-Dimethylthiazol-2-yl)-2,5-diphenyltetrazolium bromide was purchased from Calbiochem (Burlington, MA, USA).

### 4.2. Extraction

Fenugreek seed powder (500 g) was macerated with 95% ethanol (1:5) for 3 days. The extraction was repeated three times (3 × 2500 mL). The whole extract solution was filtered, with ethanol evaporated under 100 mbar at 40 °C using a rotary evaporator (Heidolph, Schwabach, Germany). The obtained extract was the oily yellowish–brown paste with 5% yield (*w*/*w*) and it was kept at 4 °C until use.

### 4.3. UHPLC Validation and Identification of Rutin in Fenugreek Extract

Rutin trihydrate, as a standard marker, was used to analyze fenugreek extract. The UHPLC method was developed and validated in terms of linearity, accuracy, precision, and sensitivity [31,32] by a Shimadzu LC-30 AD UHPLC system comprising a diode array detector SPD-M20A and an autosampler SIL-30AC. The method used a SUPLECO Titan C18 column (5 cm × 2.1 mm, 1.9 µm, SUPLECO, Burlington, VT, USA) at 30 °C and detected a wavelength of 260 nm. The mobile phase was adapted from Kenny et al. [45]. Briefly, 0.05% *v*/*v* of formic acid solution in ultrapure water (A) and 0.05% *v*/*v* of formic acid in acetonitrile (B) was prepared with a flow of 0.25 mL/min in gradient mode: the starting condition was 10% B for 1.0 min, increased to 75% B for 3.5 min, decreased to 10% B for 0.75 min, and held for 0.25 min.

### 4.4. Collagenase Assay

A collagenase assay was performed to determine anti-collagenase activity [60]. The mixture solution, containing 25 µL of 50 mM tricine buffer solution (pH 7.5), 25 µL of 2 units/mL collagenase (clostridium histolyticum type IA), and 25 µL of the sample, was prepared and incubated at room temperature for 15 min. To start the reaction, 100 µL of synthetic substrate and 2 mM of N-[3-(2-Furyl) acryloyl-Leu-Gly-Pro-Ala (FALGPA) was added into the mixture solution and incubated for 20 min. The change in absorbance of each supernatant was measured at 340 nm (Synergy H1 microplate reader) and the IC_50_ values were calculated by plotting a linear regression curve showing sample concentrations on the x-axis and percentage inhibition on the y-axis. The percentage inhibition was calculated using the following equation:

(1) $$\%\;\mathrm{Inhibition}=(\frac{Abs_{blank}-Abs_{sample}}{Abs_{blank}})\;\times \;100$$

### 4.5. Cell Culture

Human dermal fibroblast cells were purchased from the American Type Culture Collection: ATCC (PCS-201-010) and immortalized human keratinocytes (HaCaT) were purchased from Cell Lines Service, Germany (Cat. No. 300493). Cells were cultivated in Dulbecco’s modified Eagle’s media (DMEM) (Gibco, UK) supplemented with 10% FBS (Gibco, UK), 1% penicillin (100 units/mL), and streptomycin (100 µg/mL) (Gibco, St. Louis, MO, USA). Cells were incubated at 37 °C under a 5% carbon dioxide environment.

### 4.6. Collagen Content and Picrosirius Red Staining

Human dermal fibroblast cells were seeded at 2.5 × 10^4^ cells/well into 48 well-plates and incubated for 24 h. The cells were then treated with various concentrations of liponiosome without extracts (indicated as blank), liponiosome encapsulating fenugreek extract (LNF), and fenugreek extract for 7 and 14 days, with 0.005% DMSO used as a vehicle control. The media was changed every 2 days. The cells were then washed with PBS and fixed with 100 µL of 4% PFA for 10 min. Cells were washed with PBS (twice) and stained with 100 µL of 0.1% direct red 80 solutions for 10 min. After staining, 0.01 N HCl in 70% ethanol was added to wash the excess dye. Stained collagen was dissolved with 100 µL of 0.5 N NaOH and the absorbance was measured at 540 nm using a microplate reader (Synergy H1 microplate reader). The percentage of collagen content was calculated using the following equation:

(2) $$\%\;\mathrm{Collagen}\;\mathrm{content}=(\frac{Abs_{treated\;cells}}{Abs_{control\;cells}})\;\times \;100$$

### 4.7. Cell Viability Assay

Human dermal fibroblast cells were seeded at 2 × 10^4^ cells/well into 96 well-plates and incubated for 24 h. After incubation, different concentrations (0–1000 µg/mL) of the liponiosomes without extracts (blank), LNF, and fenugreek extract were added and cultivated for 24 h. Then, 100 µL of MTT (1 mg/mL) was added into each well and incubated for 4 h. DMSO was added to dissolve formazan product. The absorbance was measured at 570 nm using a microplate reader (Synergy H1 microplate reader). The percentage of cell viability was calculated using the following equation:

(3) $$\%\;\mathrm{Cell}\;\mathrm{viability}=(\frac{Abs_{treated\;cells}}{Abs_{control\;cells}})\;\times \;100$$

### 4.8. Liponiosomes Formulation

Pre-emulsification following homogenization was employed for liponiosome formulation. A phospholipid and emulsifier mixture was used to form a spherical lipid bilayer and cholesterol was used to enhance the rigidity of particles [15]. Briefly, soybean lecithin, cholesterol, and emulsifier were dispersed in propylene glycol and heated at 70–80 °C (oil phase), as shown in Table 2. Fenugreek extract was dissolved in propylene glycol/water (water phase) and heated in the same water bath. The aqueous solution of the extract was added continuously into lipid components and homogenized at 8000–10,000 rpm for 5–10 min using high-speed stirring (Heidolph Silent Crusher M, Kenilworth, NJ, USA). The mixture was cooled to 50 °C and tocopherol acetate was added and homogenized for another 10 min to obtain LNF. The morphology of LNF was observed by transmission electron microscopy (TEM, JEOL, Peabody, MA, USA), with the hydrodynamic size, polydispersity index PdI, and zeta potential investigated using dynamic light scattering (DLS, Nanosizer ZS, Malvern Instruments, Worcestershire, UK). The confirmation of liponiosome-melting behavior was investigated using differential scanning calorimetry (DSC, Mettler Toledo DSC1). The viscosity of the sample was measured by a digital Brookfield viscometer (model HBDV-III U CP). Briefly, 0.5 g of samples were used and the viscosity was examined using a CP-40 spindle, with the controlled speed at 0.5 rpm for 20 min at the ambient temperature.

To evaluate the stability of LNF, the particles were kept at 4 °C, 25 °C, and 40 °C for 3 months and the changes in particle size and morphology were examined.

Liposome and niosome encapsulation fenugreek extract (LF and NF) were formulated using a similar method as LNF. LF was prepared without the addition of sorbitan oleate into the oil phase, whereas niosome (NF) was prepared without the addition of the phospholipid component. Their physicochemical characterizations were reported in the supplementary Table S1.

### 4.9. Percentages of Encapsulation Efficiency and Bioactive Loading

Encapsulation efficiency (%EE) and bioactive loading (%BL) of LNF were calculated by determining the amount of encapsulated drug using an ultrafiltration technique. The amount of rutin was used as a standard marker. After 20 mg/mL of LNF was dissolved in DI water, it was centrifuged at 80,000 rpm for 4 h. The supernatant was collected and then the unencapsulated rutin was evaluated using an ultra-high-performance liquid chromatography technique (UHPLC) [61]. The % EE and % BL were calculated by the following equations:

(4) $$\%\;\mathrm{EE}=(\frac{Total\;rutin\;content-unencapsulated\;rutin\;content}{Total\;rutin\;content})\;\times \;100$$

(5) $$\%\;\mathrm{BL}=(\frac{Encapsulated\;rutin\;content}{Total\;rutin\;content})\;\times \;100$$

### 4.10. Franz Diffusion Cell

An in vitro permeable study of 7 mg/mL of fenugreek extract and LNF was investigated in a Franz diffusion cell system [62]. The synthetic membrane (Biomax 500 kDa ultrafiltration disc, Merck, MA, USA) was placed between a donor and a receptor compartment. Phosphate buffer saline (PBS) with a pH of 5.5 was stirred at 500 rpm (37 °C) and used as a receptor medium. Fenugreek extract and LNF were applied onto the membrane in the donor cell cap. Samples were permeated through the membrane and collected from the receptor compartment at 0, 2, 4, 6, 8, 12, and 24 h. The cumulative permeated rutin was evaluated and calculated by the UHPLC technique.

### 4.11. Porcine Skin Permeabilization

The permeation of LNF through porcine skin was visualized by imaging mass microscope (IMS, SHIMADZU, model: iMScopoe TRIO, Kyoto, Japan). The porcine skin was cut into 1.5 × 1.5 cm^2^. The rutin, fenugreek extract, and LNF were applied onto the cut porcine skin and stored at 4 °C for 24 h. The samples were cut using a cryostat, placed onto an indium tin oxide-coated glass slide, and sprayed by 9-aminoacridine matrix substance (9-AA). Samples were stored at −20 °C prior to analysis. An overlay of the optical images of the rutin was analyzed at 609.15 m/z.

### 4.12. Differential Scanning Calorimeters (DSC) Characterization

Differential scanning calorimetry (DSC) analysis was performed to determine the melting point of LNF using a differential scanning calorimeter (DSC, Mettler Toledo DSC1) [63]. After accurate weighing, the samples were placed in aluminum pans and sealed with a lid. In the scanning process, a heating rate of 10 °C was applied in the temperature range from 25 to 350 °C under a nitrogen gas purged at 40 mL/min.

### 4.13. Construction of Human Co-Cultured Skin Cells

Human dermal fibroblast cells were seeded at 8.5 × 10^4^ cells/well into 12 well culture inserts (Corning, Corning, NY, USA) and cultured for 24 h. The second and third layers of dermal fibroblast were repeatedly added onto the first layer on consecutive days. After 24 h of adding the third layer, HaCaT was then seeded at 8.5 × 10^5^ cells/well onto the formed fibroblast layers and cultured for another 24 h, prior to the experiment.

### 4.14. Investigation of Cell Viability and MMPs Secretion after UV Exposure in Co-Cultured Skin Cells

Human co-cultured skin cells were pretreated with 7 µg/mL of extract and 100 µg/mL of LNF (7 µg/mL of extract equivalence), together with 100 µg/mL of blank and 10 µg/mL of resveratrol as a positive control, for 24 h. Co-cultured skin cells were then washed twice with PBS and subjected to 5 J/cm^2^ of UVA and 30 mJ/cm^2^ of UVB irradiation (Solar Simulators, NY, USA). After UV irradiation, co-cultured skin cells were further incubated with tested samples for 24 h. Supernatant was then collected for MMP1 and MMP9 quantifications. The levels of UV-induced MMP1 and MMP9 secretions in human co-cultured skin cells were measured by enzyme-linked immunosorbent assay kits (MMP1: ab215083 and MMP9: ab100610, abcam, Cambridge, UK) according to manufacturing protocols.

The viability of human co-cultured skin cells after UV exposure was evaluated using a CellTiter-Glo luminescent cell viability assay kit (Promega, Madison, WI, USA). After 24 h of UV exposure, co-cultured skin cells were washed twice with PBS and 100 μL of Glo Lysis Buffer (Promega, Madison, WI, USA) was added into each well. After incubation for 10 min, 50 µL of CellTiter-Glo reagent was added and incubated at room temperature for 10 min. The luminescent signal was then measured using microplate luminometer (SpectraMax L, Molecular Devices).

### 4.15. Statistical Analysis

Results were represented as mean ± standard deviation (SD). Significant differences were analyzed by a Student’s *t*-test or one-way or two-way analysis of variance (ANOVA) followed by Tukey’s post hoc test (GraphPad Prism 9), with *p* values < 0.05 considered statistically significant.

## 5. Conclusions

Collectively, this research produced two alternative suggestions. Firstly, rutin was not only observed in terms of ultraviolet radiation protection and antioxidative substance, but was also used as a standard marker of chromatogram fingerprints and a key reference of anti-aging activity from fenugreek seed powder extract. Secondly, molecular mechanisms underlying ethanolic extract against aging were demonstrated. The extract showed new biological properties, such as anti-collagenase activity, inducing collagen production, and inhibiting collagen degradation, which refers to a potential alternative natural anti-aging agent for anti-aging products. Encapsulation techniques can be used to protect the bioactive compounds, chemical and physical degradation processes, and prolong their biological activity until use. This technique also reduces the toxicity of herbal extracts. In this study, formulated liponiosomes are highly stable and have a prolonged release. Nano-formulation can also enhance the potency of fenugreek extract on anti-aging properties. On the basis of this study, we suggest that LNF could potentially be applied as a promising active ingredient in the prevention of skin aging.

## Figures and Tables

**Table 1:** Parameters of method validation for Rutin detection based on AOAC guidelines.

Parameters	Results
Linear range	
Equation	Y = 3308.80x − 15,055.67
Coefficient of determination (r^2^)	0.9998
Accuracy (% Recovery)	
100, 200, 400 µg/mL	104.85, 106.16, 102.85
Precision (% RSD * of % Recovery)	
Intra-day: 100, 200, 400 µg/mL Inter-day: 100, 200, 400 µg/mL	0.99, 0.57, 0.34 1.00, 0.56, 0.24
Sensitivity	
Limit of Detection: LOD (µg/mL) Limit of Quantitation LOQ (µg/mL)	5.17 15.67

* % RSD: % Relative standard deviation.

**Table 2:** Chemical components of formulated LNF.

Chemicals	%
Part A: oil phase	
Cholesterol	5
Propylene glycol	10
Sorbitan oleate	15
Phospholipid: soybean lecithin	10
Part B: water phase	
Fenugreek extract	10
Propylene glycol	10
DI water	30
Part C: Edge activator: Tocopherol acetate	8
Part D: Preservatives	2

**Table 3:** Physicochemical properties of formulated LNF.

Parameters	Initial
Size (nm)	174.7 ± 49.2
Polydispersity index (PdI)	0.26 ± 0.04
ζ Potential (mV)	−26.0 ± 1.2
pH	6.38 ± 0.05
Viscosity (cP)	49,930 ± 46
% Encapsulation efficiency	46.6 ± 7.4
% Drug load	33.5 ± 4.0

## Data Availability

The data presented in this study are available in article and supplementary material.

## Supplementary Materials

The following supporting information can be downloaded at: https://www.mdpi.com/article/10.3390/ph15020254/s1, Figure S1: The confirmation of Rutin as a main active ingredient in Fenugreek extract using LC-MS; Table S1: Physicochemical properties of Liposome encapsulating fenugreek extract (LF) and niosome encapsulating fenugreek extract (NF).
