# Supplementary material for: Ethanolic Fenugreek Extract: Its Molecular Mechanisms against Skin Aging and the Enhanced Functions by Nanoencapsulation
Source: Pharmaceuticals (Basel). 2022 Feb 20;15(2):254. doi: 10.3390/ph15020254 (PMC8879298; doi:10.3390/ph15020254)
Supplement: Supplementary file 1 [file pharmaceuticals-15-00254-s001.zip › pharmaceuticals-1522614-supplementary.pdf]

Supplementary Data:

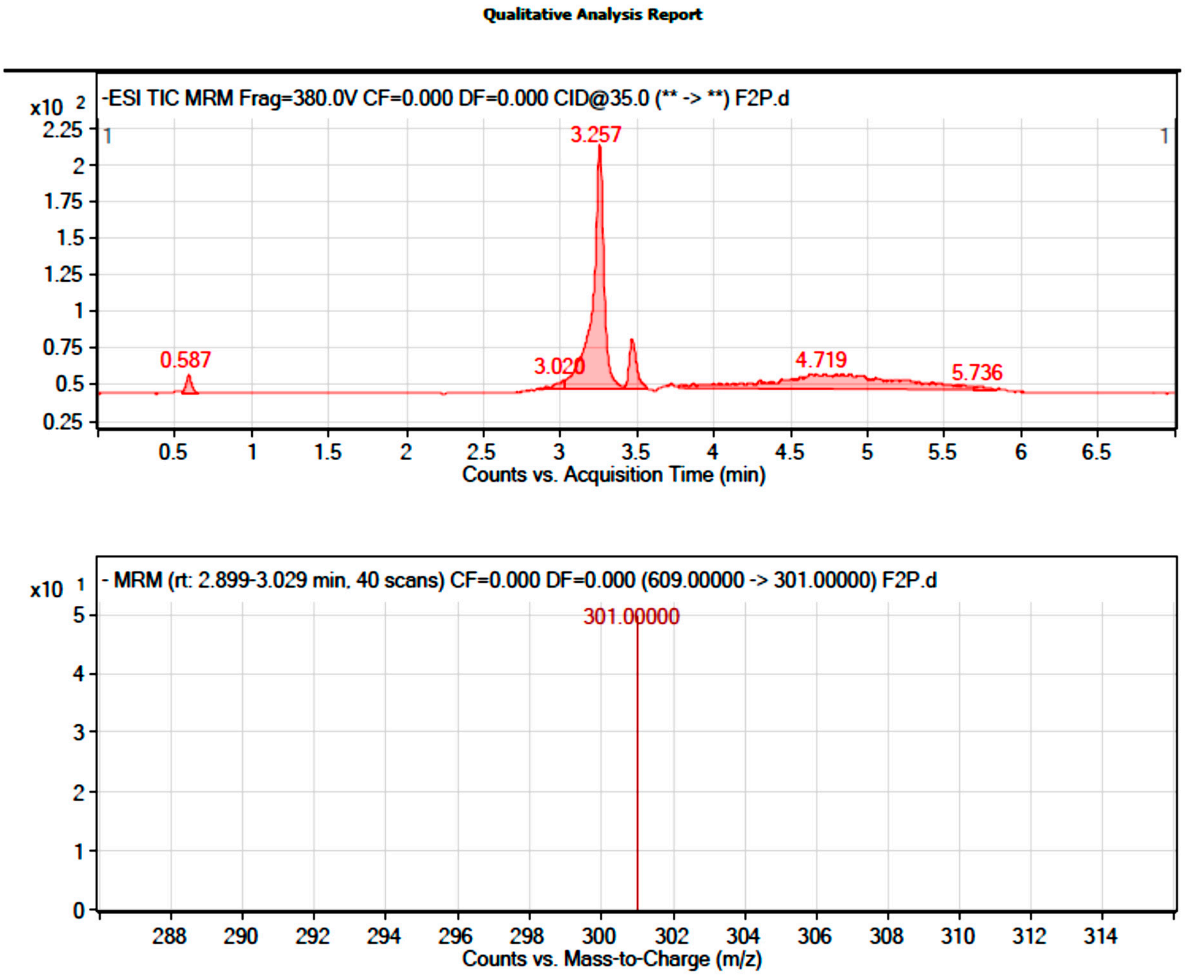

Supplementary Figure S1: LC-MS Chromatogram of fenugreek extract and the detected Ion of Rutin. Chromatographic separation was accomplished on a SUPLECO Titan C18 column (5 cm × 2.1 mm, 1.9 µm, SUPLECO, USA) at 30 °C with a 6 min gradient elution using acetonitrile and 0.05% formic acid aqueous solution as mobile phase at a flow rate of 0.25 mL min(−1). A tandem mass spectrometric detection was conducted using multiple reaction monitoring (MRM) via an electrospray ionization (ESI) source and operating in the negative ionization mode. Molecular weight of Rutin was 609 g/mol, corresponding to precursor Ion 609 (MS1 Res: Unit) and detected Product Ion 301 (MS2 Res: Unit).

Supplementary Table S1: Physicochemical properties of Liposome encapsulating fenugreek extract (LF) and niosome encapsulating fenugreek extract (NF).

Parameters	Initial			
	**Blank-LF**	**LF**	**Blank-NF**	**NF**
Size (nm)	177.33 ± 2.89	224.27 ± 10.03	89.19 ± 1.40	264.53 ± 22.86
Polydispersity index (PdI)	0.24 ± 0.02	0.25 ± 0.02	0.29 ± 0.01	0.78 ± 0.19
ζ potential (mV)	0.06 ± 0.34	−1.40 ± 0.72	−2.32 ± 0.21	−4.12 ± 0.41
